# Potential of Natural Feed Additives in Reducing Gaseous Emissions and Environmental Footprint in Rabbit Housing Systems

**DOI:** 10.3390/ani16081147

**Published:** 2026-04-09

**Authors:** Katarzyna Karpińska, Bożena Nowakowicz-Dębek, Dorota Kowalska, Paweł Bielański, Łukasz Wlazło, Mateusz Ossowski

**Affiliations:** 1 Department of Animal Hygiene and Environmental Hazards, Faculty of Animal Sciences and Bioeconomy, University of Life Sciences in Lublin, 13 Akademicka Street, 20-950 Lublin, Poland; bozena.nowakowicz@up.edu.pl (B.N.-D.); lukasz.wlazlo@up.edu.pl (Ł.W.); mateusz.ossowski@up.edu.pl (M.O.); 2 Department of Small Livestock Breeding, National Research Institute of Animal Production, Balice, 32-083 Kraków, Poland; dorota.kowalska@iz.edu.pl (D.K.); pawel.bielanski@iz.edu.pl (P.B.)

**Keywords:** natural feed additives, rabbit production, gaseous emissions, ammonia, methane, environmental footprint

## Abstract

Reducing the environmental impact of animal production is a major challenge in the context of climate change and sustainable agriculture. Although rabbit farming is generally considered less resource-intensive than other livestock systems, it still contributes to emissions of ammonia (NH_3_), hydrogen sulfide (H_2_S), and methane (CH_4_), which can negatively affect air quality and the climate. This study aimed to evaluate whether dietary supplementation with selected natural feed additives could mitigate gaseous emissions and lower the environmental footprint of rabbit production. An experimental feeding trial was conducted in which gaseous emissions from rabbit housing were monitored, and the gas composition of feces was analyzed. Emissions were quantified and expressed as carbon dioxide equivalents (CO_2_e) to allow comparative assessment of environmental impact. The inclusion of natural feed additives significantly reduced the emission of gaseous pollutants compared with the control diet, resulting in a lower calculated environmental footprint of the production system. These findings indicate that targeted modification of rabbit diets using natural feed ingredients can be an effective strategy for reducing harmful gaseous emissions and enhancing the environmental sustainability of rabbit farming.

## Simple Summary

Rabbit farming is often considered more environmentally friendly than other types of livestock production, but it still releases gases such as ammonia and methane, which can harm air quality and contribute to climate change. The aim of this study was to investigate whether adding natural substances—biochar and a plant called *Tribulus terrestris*—to rabbit diets could reduce these harmful emissions. We found that rabbits fed diets containing these natural additives, particularly biochar, produced lower levels of these gases compared with rabbits fed a standard diet. These results suggest that using natural feed additives in rabbit farming could help make animal production cleaner and more sustainable, benefiting farmers, consumers, and the environment.

## 1. Introduction

Agriculture, especially the livestock production sector, is perceived as burdening the environment and contributing to climate change. Elements counteracting climate change include measures aimed at reducing emissions of pollutants, the implementation of sustainable development, and preservation of biodiversity. New strategies are expected to include innovative measures that benefit animal welfare and at the same time reduce the environmental impact of farms [1]. These measures involve precision agriculture and digitalization, mainly in manure management, but also for reducing greenhouse gas emissions from farms with large animals. The procedures developed are extended to other species, including rabbits [2,3,4,5,6,7].

Rabbit meat production in Europe in 2023 was estimated at about 140,000 tonnes, which is more than 20% of global production. Major producers include Italy and Spain, while China remains the leading global producer. The future of rabbit meat production in Europe is influenced by several factors, including market trends, consumer preferences, agricultural policy, and regulations related to livestock farming. In recent years, rabbit meat consumption in several European countries has shown a declining trend, which is partly attributed to changing dietary habits and the increasing popularity of rabbits as companion animals. Nevertheless, rabbit meat is still valued for its high nutritional quality and relatively low environmental impact compared with other types of meat. As a result, production remains more stable in countries with a long-standing tradition of rabbit farming, such as Italy, Spain, and France. In Poland, rabbit meat continues to be perceived as a niche product with high nutritional value, and slaughter volumes have shown year-to-year variability rather than a consistent upward trend. [4,8,9,10].

According to statistical data, the share of agriculture in greenhouse gas emissions in 2022 was 0.3% for carbon dioxide (CO_2_), 58.4% for methane (CH_4_) in CO_2_ equivalent (CO_2_e), and 76.2% for nitrous oxide (N_2_O) CO_2_e. An important indicator of a farm’s environmental pressure is livestock density. For the UE, an average of 0.7 livestock units (LSU) per hectare is assumed. An increase in this indicator means an increase in the burden of pollutants from animal production on components of the environment, including soil, water, and air [11]. As a consequence of animal production, both animals and the products of their metabolism, in the form of manure and slurry, are concentrated on small units of area.

Animal excrement is a significant source of emissions of gaseous pollutants, but rational use of manure has beneficial effects by increasing the activity and diversity of soil microbes and increasing the content of organic carbon in the soil, which is beneficial for its structure and pH [5,12,13]. Calvet et al. [12] estimated that nitrogen excretion by rabbits amounts to about 58% of its total intake, and the production of 1 kg of meat introduces 40 g of nitrogen into the environment [12]. However, this is significantly less than in the case of pig or poultry farming. Cesari et al. [14] estimated the environmental burden of rabbit production at 3.86 kg of CO_2_ equivalent/kg body weight. According to the authors, this is higher than for broilers and similar to the value for pork production. However, the environmental burden in commercial rabbit farming depends on the mortality rate, feed conversion, amount of meat produced, and manure management system [4,14]. According to the authors, irrespective of the method of applying manure to the soil, significant losses in ammonia and carbon dioxide levels are noted. Dinuccio [15] indicates that the problem of emissions in rabbit farming does not only concern manure. Estellés et al. [16] report that carbon dioxide accounts for a large portion of pollutant emissions, at a level of 1.98 ± 0.72 L h 1^−1^, at the same time pointing out that it is correlated with the rabbits’ weight and activity [16].

Rabbit farming, although generally considered relatively environmentally friendly compared with other livestock production systems, still generates a measurable environmental impact, mainly through greenhouse gas emissions and nutrient excretion [14,17]. Rabbits produce substantially lower amounts of methane than other herbivores and farm animals, as confirmed by both in vivo measurements and manure emission analyses. Nevertheless, due to the large global population of rabbits, their contribution to greenhouse gas emissions remains measurable, albeit clearly lower than that of ruminants [18,19]. The low level of methanogenesis in rabbits is primarily attributed to the specific characteristics of the caecal microbiota, which is dominated by fibrolytic bacteria with limited hydrogen production and by acetogenic microorganisms that compete with methanogens for hydrogen, thereby restricting methane formation. However, fermentative processes and potential methane production in the caecum are strongly diet dependent, as different sources of fiber and starch influence the volatile fatty acid profile and the course of caecal fermentation [20,21]. Moreover, methanogenic activity increases gradually with age and the transition to solid feed, although it remains low compared with ruminant species [22].

In the face of increasing environmental demands and the need to reduce emissions of gaseous pollutants and the carbon footprint of animal production, the search for innovative and sustainable feeding solutions is becoming a key challenge for contemporary live-stock farming. The greatest focus is placed on large animals, especially cows, due to their significant contribution to greenhouse gas emissions [6,23,24,25]. Relative to large animals, rabbit farming accounts for a small part of animal production in Europe, but prospects for the coming years assume the possibility of rapid growth in the farmed rabbit population. These animals’ low requirements, adaptability to their environment, and better feed absorption enable the development of rabbit production in many countries. Moreover, the nutritional and dietetic value of rabbit meat is increasing interest in this species [1,10,26]. Given the socioeconomic changes in Europe resulting from the introduction of restrictions on livestock farming and climate policy, rabbit farming may be an effective and sustainable alternative in the production of animal protein. Due to their low requirements, rabbits can be kept in extensive systems (backyard farming) and in areas with poor feed resources [14,27]. Rabbits are superior to other livestock species in terms of feed efficiency, which is of key importance for the environmental economy and the economic outcomes of livestock farming [28]. Rabbit farming allows animal production to be maintained in areas with harsh climate and agricultural conditions and supports short supply chains in rural areas, helping to prevent their social exclusion [17,29]. In countries with developed economies, research is conducted on optimal rabbit production systems maintaining a high feed conversion rate and minimizing the environmental burden [14,30]. Researchers indicate that rabbit meat production burdens the environment with nitrogen and phosphorus significantly less (by about 10%) than other livestock species [14]. The search for strategies utilizing natural feed additives for rabbits, as in the case of other animals, may additionally reduce the environmental pressure of these farms, in particular by reducing emissions of gases, including greenhouse gases. Feed additives used in livestock animals such as pigs or cows are based on vermiculite (zeolite), aluminosilicates, and recently also biochar. This makes it possible to reduce pollution of the air in livestock buildings and to reduce emissions from manure without compromising the animals’ health or production efficiency.

The environmental footprint of rabbit meat production is largely determined by feed composition, as demonstrated in rabbits and other livestock species [31]. The search for alternative protein sources and functional feed additives is therefore essential for the sustainable development of this production system [23]. Previous studies have shown that agro-industrial by-products and plant-derived additives can influence nitrogen utilization and gaseous emissions in rabbits [31]. In particular, saponins present in *Tribulus terrestris* may reduce ammonia formation by inhibiting urease activity in the digestive tract, thereby limiting NH_3_ emissions and improving nitrogen retention [5,32,33,34,35]. Similarly, biochar-based materials have been reported to adsorb nitrogenous compounds and modify microbial activity, which may reduce the emission of ammonia and methane from animal manure [36,37].

Despite these findings, the effectiveness of natural feed additives in mitigating gaseous emissions under practical rabbit farming conditions remains insufficiently documented, and the underlying mechanisms are not fully understood. Moreover, although various emission-reduction strategies have been proposed, gaseous emissions from livestock farms remain a persistent environmental problem [38,39,40], highlighting the need for further targeted research.

Therefore, the present study was designed to test the hypothesis that dietary supplementation with biochar and *Tribulus terrestris* can reduce the emission of ammonia, hydrogen sulfide, and methane from rabbit production systems by improving nitrogen utilization and altering microbial processes in the digestive tract and feces. Specifically, we hypothesized that these natural additives would lead to lower gaseous emissions and, consequently, a reduced carbon footprint of rabbit meat production.

## 2. Materials and Methods

### 2.1. Ethical Approval

All research methods were approved by the Local Ethics Committee for Experiments on Animals of the University of Life Sciences in Lublin, Poland (Resolution No. 62/2023 of 11 September 2023), and the study was carried out in compliance with the guidelines of the European Union [34].

### 2.2. Experimental Design

A total of 80 New Zealand White and Popielno White rabbits were assigned to four groups—a control and three experimental groups. The experiment began at 35 days of age and continued until day 120. There were 20 animals in each group, matched for body weight (close to the average) and sex ratio. The mean body weight on day 35 was as follows: 826.0 g in group C, 834.7 g in group D1, 833.3 g in group D2, and 834.0 g in group D3. Rabbits of the same sex were housed two per cage, resulting in 10 replicates per treatment group. Throughout the experimental period, rabbits had ad libitum access to feed and water. Animals were housed in two-tier commercial wire-mesh cages equipped with standard feeders and nipple drinkers. Each cage measured 80 × 60 × 40 cm and was located in a closed, climate-controlled building fitted with a chimney and a mechanical exhaust ventilation system, in accordance with current animal welfare regulations. After being assigned to groups, they were kept in the same herd management system (housing and feeding) for a one-week adaptation period, after which the animals in each experimental group received mixtures of the experimental factors together with their feed.

### 2.3. Animal Diets

The basic feed mixture used in this study was previously described by Wlazło [41]; it was used in two independent experiments with different designs. The diet supplied about 168 g/kg crude protein, 10.3–10.5 MJ/kg metabolizable energy, and 130 g/kg crude fiber. It also contained roughly 34 g/kg ether extract and 73 g/kg crude ash, with stable mineral levels, including calcium at around 8.3 g/kg and phosphorus at 7.1 g/kg. The amino-acid profile remained consistent, with lysine decreasing slightly from 7.6 to 6.7 g/kg across treatments.

The animals in the control group (C) received a basal diet. The animals in the experimental groups received the same diet but with the following natural additives:

Group D1—0.25% biochar in the daily diet;

Group D2—0.25% biochar and 0.1% *Tribulus terrestris* (TT) in the daily diet;

Group D3—0.1% *Tribulus terrestris* (TT) in the daily diet.

The biochar was produced according to a standardized procedure by an external company (Feinor sp. z o.o., Poprężniki, Poland) and is protected by patent rights. The biochar composition was as follows: crude fiber 77–80%, crude ash 25–28%; it did not contain crude protein, lysine, methionine, sodium, crude oils, or fats, and contained only a small amount of volatile matter (V_daf_ = 5.3%). *Tribulus terrestris* was used in powdered extract form and, similarly to biochar, was incorporated into the feed during the pelleting process. The TT extract was obtained as a commercial plant supplement. According to the manufacturer, the dried fruit extract of *T. terrestris* contains 90% dry matter per 100 g of product, with crude protein < 1 g, fat < 1 g, fiber 5–8 g (extraction residue), and ash 1–2 g. The extract contains saponins, including protodioscin (47%) and dioscin (14%). According to the scientific literature, *T. terrestris* fruit also contains minerals.

### 2.4. Measurements and Sampling

The study was conducted from September to December and involved a comprehensive assessment of the effects of dietary supplementation on the microclimate of rabbit housing, emissions of gaseous pollutants, and the comparative environmental footprint of each experimental group. To evaluate air quality in the rearing environment, concentrations of ammonia (NH_3_), hydrogen sulfide (H_2_S), and methane (CH_4_), as well as levels of suspended particulate matter (PM2.5 and PM10), during two measurement periods, each consisting of three measurement sessions conducted at approximately 13-day intervals. The values presented in the Results section represent the mean of measurements obtained within these two periods. The reduction in released gaseous pollutants for each group in each stage was calculated according to Equation [42]:

R*e* = 100% − [(100%*CG*)/*CC*],

where

R*e*—gas reduction (%);

*CG*—amount of gas released in the test group on a given test day;

*CC*—amount of gas released in the control group on the same day.

Measurements were performed using an Atmon FL measurement chamber (Nanosens, Wysogotowo, Poland), equipped with modules for detecting ammonia (0–100 ppm), hydrogen sulfide (0–50 ppm), and particulate matter (0–999 µg/m^3^). The spatial distribution of NH_3_ and H_2_S concentrations within the housing facility was visualized using isoline maps, allowing for a detailed assessment of emission patterns. Simultaneously, microclimatic conditions inside the rabbit housing, including temperature and relative humidity, were continuously monitored using a wireless Efento system (Efento sp. z o.o., Krakow, Poland).

In addition, fecal samples were collected at the beginning of the experiment from all experimental groups and placed in a controlled measurement chamber for the analysis of NH_3_, CH_4_, and H_2_S concentrations, as well as monitoring of oxygen (O_2_) levels, using an integrated system with a Fresenius GA 220 Analyser (Fresenius Umwelttechnik GmbH., Herten, Germany). Gas measurements in the chambers were conducted over a four-week period, with pollutant concentrations recorded at the end of each week. While the use of controlled measurement chambers allows for standardized and comparative assessment of gaseous emissions from fecal material, it does not fully replicate the conditions present in commercial housing systems. Therefore, the results obtained should be interpreted as indicative of potential emission trends rather than direct representations of farm-scale emissions.

For the environmental assessment, a simplified and comparative approach was adopted, focusing exclusively on gaseous emissions originating from animal metabolism and manure decomposition under controlled farm conditions. Other components of the production system, such as feed production, water use, and energy consumption, were excluded from the analysis, as these factors were identical across all dietary treatments and did not influence relative differences between groups. Measured concentrations of CH_4_, NH_3_, H_2_S, N_2_O were used as the basis for estimating emission intensity. These concentrations were further converted into emission fluxes by incorporating the ventilation rate of the housing system. A constant airflow rate was assumed for all experimental groups, corresponding to the standard operating conditions of the facility. Concentration values were converted into mass-based emissions using the ventilation flow, which enabled the estimation of emission rates under uniform environmental conditions. To enable comparison of climate-related impacts, greenhouse gas emissions were expressed as carbon dioxide equivalents (CO_2_e) using Global Warming Potential values from IPCC AR6 (GWP100: CH_4_ = 27; N_2_O = 273). Nitrous oxide (N_2_O) emissions were directly measured in the study and included in the calculation of CO_2_e based on the measured emission values.

Accordingly, the calculated CO_2_e values should be interpreted as a comparative indicator of emission-related environmental impact between dietary treatments rather than as a complete product carbon footprint compliant with ISO 14067 [43]. This approach nevertheless allows for a robust evaluation of the relative effectiveness of natural feed additives in mitigating emission-related environmental pressure under uniform experimental conditions, which constituted the primary objective of the study [44].

### 2.5. Statistical Analysis

Statistical analyses were performed using Statistica software version 13.3 (StatSoft Inc., Tulsa, OK, USA). Results are presented as arithmetic means (M) with the standard error of the mean (SEM). Prior to analysis, the normality of residuals distribution was assessed using the Shapiro–Wilk test, and homogeneity of variances was verified using Levene’s test.

Differences between dietary treatments (C, D1, D2, D3) were evaluated using one-way analysis of variance (ANOVA). When significant effects were detected, post hoc comparisons were performed using Tukey’s test. Differences were considered statistically significant at *p* < 0.05. Means within columns marked with different superscript letters (a, b, c, d) indicate statistically significant differences between groups.

## 3. Results

### 3.1. Air Monitoring in the Hutch

Microclimatic conditions in the hutch were monitored during the experiment. The temperature ranged from 15.1 to 20.1 °C (average 17.8 °C), and the relative humidity was 37–65% RH (average 47.7% RH)—typical conditions for rabbit production. The ammonia concentration in the air increased with the animals’ growth (Table 1). The highest NH_3_ level was recorded during the second measurement session in the control group (11.89 ppm) and was significantly higher than in the experimental groups (*p* < 0.05), while the lowest concentration was recorded in the first measurement session in group D1 (3.1 ppm). This value was significantly lower than in the other groups (Table 1). The presence of hydrogen sulfide was not identified in the hutch in either of the measurements using the technique employed in the study (Table 1).

**Figure 1:**
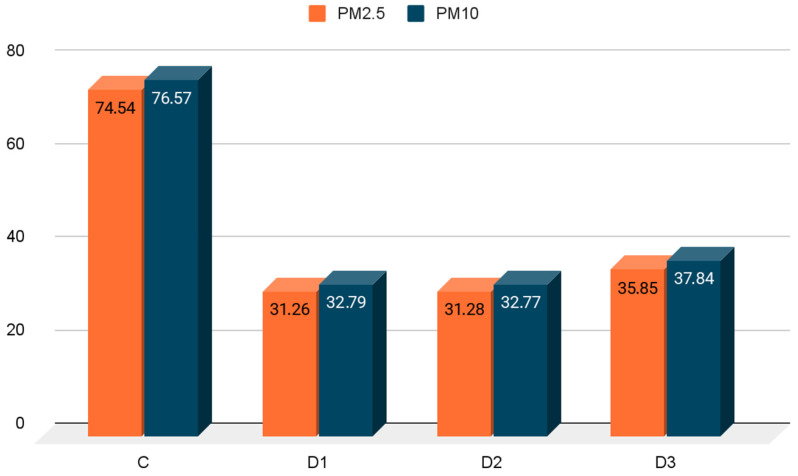
Mean concentrations of particulate matter (PM_2_._5_ and PM_10_) during the entire study period, presented as group means. [µg/m^3^].

The average PM2.5 concentration in the air of the hutch was 49.83 µg/m^3^. Its highest level was recorded during the first measurement session in the control group (84.39 µg/m^3^) and was statistically significantly higher than in the experimental groups (Table 2). The lowest PM2.5 level during this period was recorded in group D1 (19.13 µg/m^3^) and was statistically significant compared to the control group and experimental group D3 (36.34 µg/m^3^). During the second measurement session, the PM2.5 level was slightly higher in all groups, but the highest level was obtained in the control group (64.68 µg/m^3^) and was significantly higher than in the experimental groups (*p* < 0.05). The lowest PM2.5 level at this time was obtained in group D3 (35.35 µg/m^3^). Similar changes in air quality were obtained for PM10 (Table 2). The PM10 concentration was highest in the control group (86.46 µg/m^3^) during the first measurement session, and it was significantly higher than in group D1 (20.20 µg/m^3^), D2 (22.59 µg/m^3^), and D3 (38.33 µg/m^3^), at *p* < 0.05. During the second measurement, there was a slight increase in the dust concentration in groups D1 (45.38 µg/m^3^) and D2 (45.38 µg/m^3^), while a slight decrease was noted in groups D3 (37.35 µg/m^3^) and C (66.68 µg/m^3^). These values were statistically significant (*p* < 0.05). The mean PM2.5 and PM10 concentrations during the study period are presented in a graph (Figure 1). Their levels were highest in the control group (PM2.5—74.54 µg/m^3^, PM10—76.57 µg/m^3^) and lowest in group D1 (PM2.5—31.26 µg/m^3^, PM10—32.79 µg/m^3^) and D2 (PM2.5—31.28 µg/m^3^, PM10—32.77 µg/m^3^). A significant reduction in dust levels was obtained in the experimental groups.

**Figure 2:**
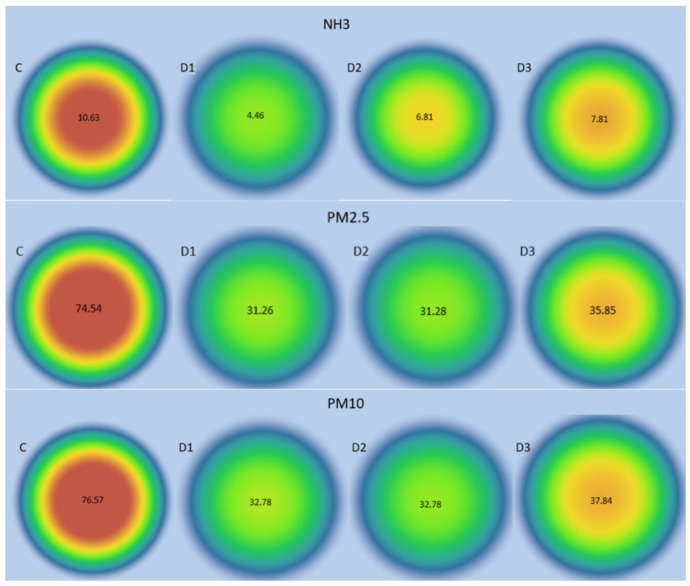
Visualization of the distribution of gaseous pollutants on the rabbit farm (NH_3_ ppm; PM2.5 µg/m^3^, PM10 µg/m^3^)—graphic distribution of mean concentrations in each group. Colors ranging from red (highest values in µg/m^3^) to blue illustrate the distribution of gaseous pollutants and particulates between the control and experimental groups.

The concentrations of all pollutants were highest in the control group. The visible differences in color correspond to differences in the intensity of pollutants between groups. The use of feed supplements reduced emissions of NH_3_ and particulates (PM2.5 and PM10), potentially improving the environmental conditions in the hutch (Figure 2).

### 3.2. Emissions of Gaseous Pollutants from Rabbit Feces

**Figure 3:**
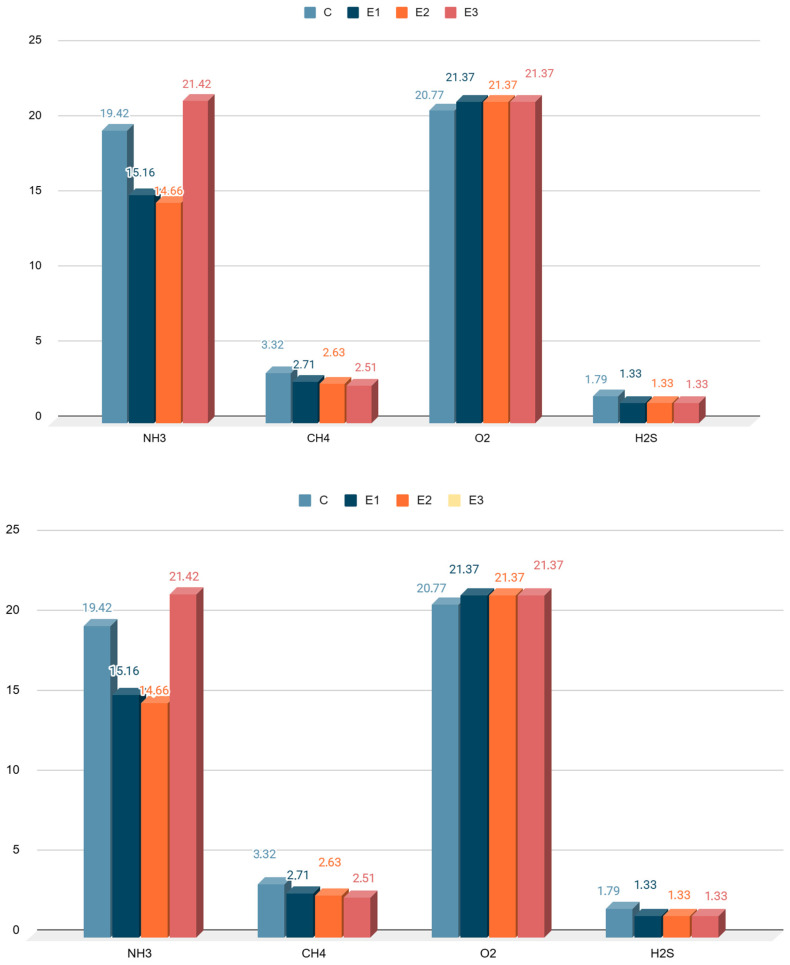
Mean concentrations of gaseous pollutants (NH_3_, CH_4_, H_2_S) released from rabbit feces [ppm], together with oxygen concentration [% vol.] in the measurement chambers during the experiment. NH_3_ levels were highest in D3 and C and much lower in D1 and D2. CH_4_ concentrations were relatively low in all groups, with slight differences. The O_2_ level was fairly stable, with a slight difference between group C and the experimental groups. H_2_S emissions were highest in group C and lower in groups D1, D2, and D3.

**Figure 4:**
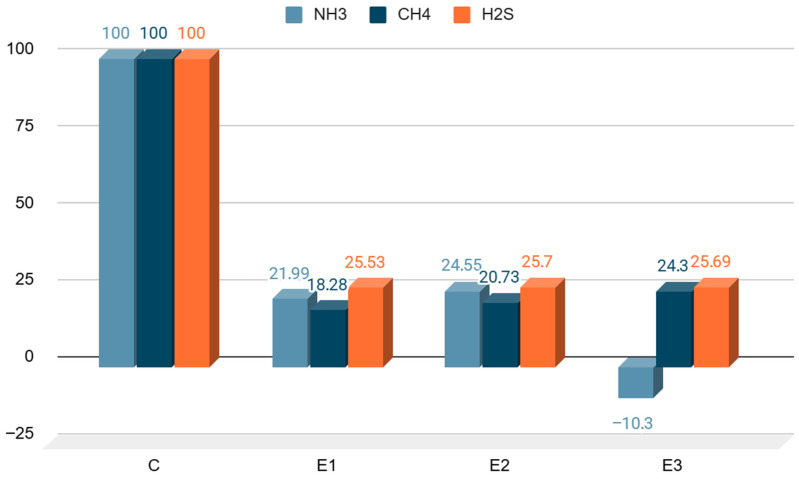
Percentage reductions in gaseous pollutants NH_3_, CH_4_, and H_2_S in each group (%). Control group values were adopted as the baseline (100%).

The results for emissions of gaseous pollutants released from feces placed in the measurement chambers are presented in Table 3. Analysis of the data showed significant differences in the concentrations of ammonia (NH_3_), methane (CH_4_), and hydrogen sulfide (H_2_S) between sampling times. The concentrations of all pollutants were highest in the first week of the study and decreased significantly in subsequent weeks. These values indicate an initial intensification of the decomposition of nitrogen compounds in all groups. The highest NH_3_ concentration was recorded in the control group in the first period of the experiment (85.36 ppm), while the lowest was recorded in group D1 (26.66 ppm). These values were statistically significant compared to the other experimental groups (*p* < 0.05). The methane concentration in the control group in the first week was significantly higher than in group D3 and amounted to 4.44 ppm. The concentration of this gas decreased in subsequent weeks, and this significance persisted at *p* < 0.05. The hydrogen sulfide concentration was highest in the first week of the study in the control group (3.24 ppm), and it was statistically significantly higher than in all experimental groups. This tendency, despite the decrease in H_2_S concentrations, persisted until the end of the experiment. Significant differences were shown in the average concentrations of gaseous pollutants from the measurement chambers (Figure 3). The average NH_3_ concentrations in groups D1 and D2 were significantly lower than in groups C and D3. The methane concentration during the entire study period was significantly higher in group C, amounting to 3.32 ppm. The oxygen concentration ranged from 20.77% vol. in group C to 21.37% vol. in the experimental groups. In the case of H_2_S, the average levels ranged from 1.33 ppm in the experimental groups to 1.79 ppm in the control group. These values were statistically significant at *p* < 0.05. The use of feed additives for rabbits reduced emissions of pollutants, which was confirmed by calculating the size of the reductions (Figure 4). The greatest reductions in NH_3_ were obtained using biochar and biochar in combination with *Tribulus terrestris* in groups D1 and D2 and amounted to 21.99% and 24.55%, respectively. A similar trend was noted for CH_4_ in these groups; the reduction was 18.28% in group D1 and 20.87% in group D2.

A reduction in all gaseous pollutants was observed in groups D1 and D2, with similar levels. A different trend was noted in group D3—a reduction in CH_4_ and H_2_S and an increase in NH_3_. The results indicate varied effectiveness of the methods for reducing emissions.

### 3.3. Estimation of Environmental Footprint

Environmental footprint calculations showed varied levels of CO_2_e emissions depending on the feed additives used. Analysis of the results made it possible to assess the potential environmental benefits of optimizing the feed composition, which can be significant for sustainable animal production. The most effective additive for reducing the environmental footprint was biochar (0.13 kg CO_2_e). Supplementation with biochar in combination with *Tribulus* (D2) and *Tribulus* alone (D3) were less effective: 0.20 kg CO_2_e and 0.23 kg CO_2_e, respectively.

**Figure 5:**
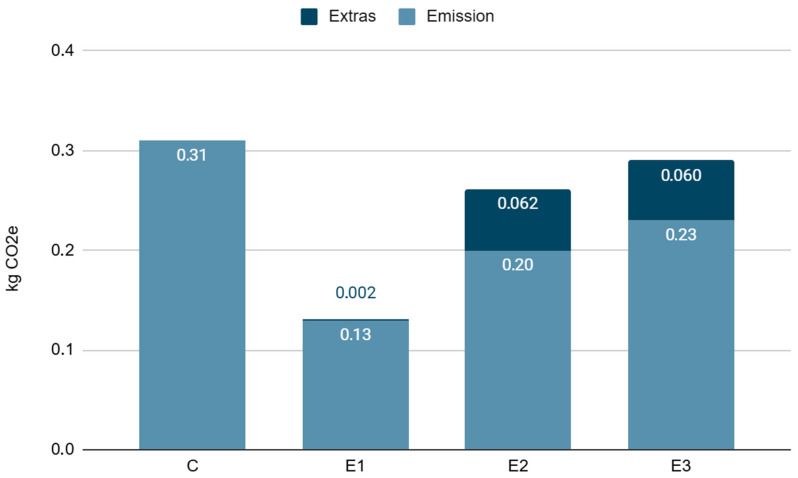
Environmental footprint expressed as kg CO_2_e in each experimental group, calculated based on emissions of CH_4_ and N_2_O as well as contributions from feed additives.

The environmental footprint analysis showed significant differences between experimental groups, with the highest CO_2_ equivalent (CO_2_e) noted for the control group. A significant reduction in the environmental footprint was obtained in the groups whose diet was supplemented with natural additives, even after taking into account the environmental cost associated with their production and transport. The lower CO_2_e values in the experimental groups indicate that the additives used can reduce emissions of gases, including greenhouse gases associated with digestion and excrement decomposition (Figure 5).

## 4. Discussion

Researchers indicate that rabbit meat production burdens the environment with nitrogen and phosphorus significantly less (by about 10%) than other livestock species [14]. The search for strategies utilizing natural feed additives for rabbits, as in the case of other animals, may additionally reduce the environmental pressure of these farms, in particular by reducing emissions of gases, including greenhouse gases. Feed additives used in livestock animals such as pigs or cows are based on vermiculite (zeolite), aluminosilicates, and recently also biochar. This makes it possible to reduce pollution of the air in livestock buildings and to reduce emissions from manure without compromising the animals’ health or production efficiency. Similar strategies can potentially be tested for rabbits in order to mitigate pollution of the environment [3,6,38,45,46,47]. Recent years have seen great interest in biochar [26,46,48,49,50]. Biochar is an environmentally friendly material arising in the process of pyrolysis. Its mechanism of action depends on the conditions determining its transformations in the environment. Its porous structure promotes chemical adsorption of contaminants, including ammonia and microorganisms. In the present study, a significant reduction in the NH_3_ level was obtained in group D1, in which biochar was added to the feed, both in the air of the hutch and during the storage of feces in measurement chambers. At the same time, physical adsorption increases the bioavailability of trace elements (e.g., Fe, Zn, Se, and Cu), creating a favorable environment for the development of microorganisms [50]. As no studies have previously been conducted on the use of biochar in the diet of rabbits, the results of the present study can only be compared to research using other species. According to Bogolyubova et al. [51], supplementation with activated carbon in small ruminants in the amount of 1 g per 10 kg live weight improves feed digestion and increases the nitrogen utilization coefficient, thus reducing the environmental burden. Similar results were obtained by Prasai et al. [48] in an experiment in poultry. Supplementation with biochar as well as with zeolite reduced ammonia emissions and the level of nitrogen in the feces compared to the control group. The effectiveness of biochar was found to depend on the material used to produce it and on its level of inclusion in the feed. It was estimated that the optimal diet for broilers should contain 1% biochar. In the present study, lower levels of biochar supplementation were adopted and proved to be effective and economically justified. According to Prasai et al. [52], increasing biochar supplementation even up to 2% can promote longer retention of food in the intestines. Slowing down the passage of feed through the digestive tract can lead to efficient utilization of nutrients and in consequence reduce contamination of the environment. This is confirmed by the feed conversion ratio in poultry, which was higher when the diet was supplemented with 1% biochar, even in comparison to bentonite. The use of this additive in poultry significantly reduced ammonia concentrations at bird head height while at the same time reducing N in the excreta, which reduces ammonia emissions from the excreta [52]. Covali et al. [49] used biochar to reduce NH_3_ emissions from cattle slurry. They obtained the best results using acidified biochar; after just two days, the level of NH_3_-N was significantly lower than in the control group without acidified biochar. These effects were not observed when untreated biochar was used, and the reduction in NH_3_ was short-lived. The authors recommend using acidified biochar as an effective agent for reducing emissions during storage of slurry digestate [49]. They indicate that an important factor is the pH of the solution, which regulates the conversion rate, and that aeration increases the rate of the NH_4_^+^-N to NH_3_-N process. Mixing slurry with biochar, as well as surface application of biochar, reduced ammonia emissions, and the mechanism differed somewhat in each case [49]. Similar observations were made in the present study in group D1, in which the diet was supplemented with biochar. Cai et al. [53] report that biochar can also be used to produce methane because it can be an electron carrier between bacteria and archaea. The efficiency of this process depends on its inclusion level in the feed, the pH, and the reaction environment. This may ultimately be one of the strategies to be used in practice. According to Biagini et al. [36], the use of biopolymers from urban gardening waste may be one of the systems leading to a reduction in emissions of pollutants from rabbit farming. Manure from supplemented animals reduced ammonia emissions relative to the control group by as much as 30% and methane by 25%. These values were slightly higher than those obtained in the present study in measurement chambers. A 33% reduction in ammonia emission from rabbit litter was obtained by adding calcium superphosphate to the litter. No differences were obtained for other gases [54]. The authors observed that the ammonia concentration in the air increased with the age of the rabbits, which is confirmed in the present study, in which a significant increase in pollutants in the air of the hutch was recorded in the second sample. Care for the environment requires the development of a sustainable production strategy that minimizes the environmental impact while maintaining production parameters. Hence it is worth searching for and applying natural feed additives for rabbits which are in line with this clean production and whose availability is not threatened by climate change [55,56]. In many countries, these may be plant preparations, i.e., phytobiotics containing saponins, which are known as organic substances reducing the excretion of metabolites into the environment. The plant best known for its content of steroidal saponins is *Yucca schidigera*, which inhibits methanogen populations in the rumen, reduces methane levels, and inhibits microbial urease, leading to a reduction in the ruminal ammonia concentration [55,57]. Saponins contained in herbs and extracts administered to small ruminants did not reduce the ammonia concentration, but improved the lipid profile. Rapid protein digestion in animals leads to excessive ammonia production and its removal from the body [58]. Supplementation of rabbit diets with *Yucca schidigera* preparations does not always directly reduce ammonia concentrations in their housing. In animals receiving 5 g of yucca extract per 100 kg of feed, a beneficial effect on caecal fermentation was observed, manifested as an increase in apparent protein and fat digestibility compared to the control group [59].

In the present study, *Tribulus terrestris* containing saponins was used. Feng et al. [59] reported that saponins from *Tribulus terrestris* (GSTT) reduced methane production in vitro by 23.43–25.30% compared to the control group. According to Hamzah and AL-Musawi [60], the presence of this phytobiotic in the diet of rabbits can significantly improve the serum lipid profile, reduce cell surface damage, and improve endothelial dysfunction resulting from hyperlipidaemia. However, no studies have been conducted on their use to reduce gaseous pollutants. The average ammonia concentration in the air of the rabbit hutch following supplementation with *Tribulus terrestris* and biochar (D2) was 6.80 ppm, which was significantly lower than in the control group. When *Tribulus terrestris* alone was added to the diet (D3), the ammonia level in the air was slightly higher (7.81 ppm), but still lower than in the control group. This indicates that the phytobiotic has lower reducing properties than biochar. The porous structure of biochar supports the effects of saponins, resulting in a lower NH_3_ concentration. This significant reduction in ammonia in the rabbits in group D2 was most likely due to the supporting effect of biochar on the activity of urea cycle enzymes, contributing to its conversion to urea and excretion from the body. NH_3_ emission in the measurement chamber was highest in group D3. The assumed mechanism of supporting detoxification pathways with *Tribulus terrestris*, i.e., improvement of nutrient absorption at the intestinal level by preventing oxidative damage and inhibiting the growth of pathogenic or opportunistic microbes, has proven ineffective [61,62,63]. A reduced methane concentration was noted in the measurement chambers in group D3, probably due to the effect of the phytobiotic on the microbiome (the composition of the intestinal microflora), which indirectly limits the growth of methanogens [61,64]. According to Amirshekari et al. [65], *Tribulus terrestris* regulates metabolic homeostasis in the body and the balance of the intestinal microbiota in favor of an ecological balance of microorganisms. The interpretation of the obtained results should be considered in the context of caecal fermentation physiology, which in rabbits represents the primary site of microbial transformations, including nitrogen metabolism. Microorganisms inhabiting this part of the digestive tract utilize nitrogen both in the form of ammonia and other non-protein nitrogen compounds, and its concentration reflects the balance between production processes and its reutilization in microbial protein synthesis [66]. In practice, this means that NH_3_ emission is not solely the result of its physical release, but rather reflects the balance between proteolysis and deamination processes and the ability of the microbiota to incorporate nitrogen into microbial biomass. Changes in this balance, resulting from the action of feed additives, may lead to significant differences in ammonia emissions. In the case of biochar, the most plausible mechanism of action is physicochemical. Due to its highly developed porous structure and the presence of active surfaces, this material can adsorb ammonium ions (NH_4_^+^), thereby limiting their conversion into volatile ammonia [67]. As a result, the pool of nitrogen available for emission is reduced. In turn, the effects of *Tribulus terrestris* can be primarily attributed to its influence on the gut microbiota. Saponins are compounds capable of interacting with microbial cell membranes, which may lead to changes in microbial population structure and fermentation pathways [68]. In vitro studies have shown that saponins derived from this plant can reduce methane production [59], suggesting their potential impact on methanogenic microorganisms. At the same time, there is no direct evidence that *Tribulus terrestris* inhibits urease activity under in vivo conditions in rabbits. Therefore, it is more justified to assume that its effect on NH_3_ levels is indirect, resulting from changes in the microbiome and fermentation processes rather than from direct interaction with specific enzymes. Considering the above mechanisms, the greatest reduction in ammonia observed in group D2 can be interpreted as a result of the combined action of both additives. *Tribulus terrestris* may have reduced NH_3_ formation through modulation of the microbiota, while biochar decreased its availability for emission due to its sorptive properties.

Confirmation of these mechanisms, however, requires further research. Another significant pollutant in animal production, released during the decomposition of organic matter, is hydrogen sulfide. This process depends on the protein content in the feces, the amount of sulfur-reducing bacteria, and the reaction conditions. Due to potential irritation of the respiratory tract, the acceptable concentration of this gas in livestock buildings has been estimated at 5 ppm. Significant levels of hydrogen sulfide are released during the decomposition of cow and pig manure [69]. The authors noted the highest rate of emission of this gas from cow manure in the first 24 h of their experiment (~0.168 mg/h), followed by a slight decrease. As the manure temperature decreased, the emission rate decreased. The use of natural additives to manure in ex situ conditions did not lead to a reduction in H_2_S [5]. However, the authors obtained a significant decrease in methane emissions (~50%) in these conditions. In the present study, only in group D1 were trace amounts of hydrogen sulfide detected in the measurement chambers.

According to Chen et al. [70], in addition to reducing emissions, the use of biochar for manure composting can have agronomic effects by improving the nitrogen and carbon cycle. Adopting this strategy leads to sustainable development of animal and crop production. Cesari et al. [14] assessed the environment impact of the rabbit production system, taking into account the partial life cycle, from cradle to slaughter-house. The authors obtained greenhouse gas emissions in a range of 3.78–4.04 kg CO_2_ equivalent per kg live weight for the strategies adopted. The levels were comparable with pig and poultry fattening. Feed conversion and deaths in rabbits are closely linked to sustainable development policy. Another important factor influencing the environmental performance of rabbit production is farm management, including feeding strategy and hygiene, whose optimization can substantially improve environmental efficiency [40]. In environmental research, it is also essential to identify production “hotspots”, i.e., stages that contribute most to greenhouse gas emissions. Accordingly, the present study focused on gaseous pollutant emissions, which remain a key challenge in livestock farming. The results indicate that the use of feed additives, despite the additional environmental footprint associated with their production, can reduce emissions to an extent that justifies their application in animal nutrition.

Nevertheless, several limitations of the present study should be acknowledged. The biochar applied in this experiment was produced from beechwood under controlled technological conditions and represents only one specific type of biochar. Its physicochemical properties and biological activity are known to be strongly influenced by the origin of the feedstock and pyrolysis parameters, including temperature and residence time. Therefore, the observed effects cannot be directly extrapolated to biochars derived from other raw materials or produced under different conditions. Future studies should compare biochars of diverse origins to better elucidate structure–function relationships and optimize their use in animal feeding. Similarly, the biological activity of *Tribulus terrestris* is primarily associated with its saponin content, which may vary considerably depending on plant origin, growing conditions, harvesting time, processing method, and dosage. Such variability may influence the consistency and magnitude of its effects on metabolism, gut fermentation, and gaseous emissions. Further research is therefore needed to characterize the phytochemical profile of *Tribulus terrestris* preparations and to determine optimal inclusion rates and supplementation periods under different production conditions.

## 5. Conclusions

In light of the need to reduce emissions of gaseous pollutants in animal production, rabbit farming is a promising alternative with a relatively small environmental impact. In summary, this study tested the hypothesis that dietary supplementation of rabbits with selected natural feed additives, including biochar and *Tribulus terrestris*, can reduce gaseous emissions associated with rabbit production. The results demonstrated that the applied dietary treatments significantly reduced the concentrations of ammonia, hydrogen sulfide, and methane compared with the control group, leading to a lower CO_2_-equivalent emission indicator under the applied experimental conditions. Additives of biochar additionally increases the amount of carbon in manure, which contributes to its sequestration in soil when rabbit manure is used in agriculture. These findings indicate that intentional modification of dietary composition could help alleviate environmental pressures related to emissions in rabbit farming. However, the practical implementation of such feeding strategies requires further research to confirm their impact on production performance and animal health, as well as to assess the agronomic value of manure from rabbits fed biochar-supplemented diets.

## Figures and Tables

**Table 1:** Concentrations of ammonia (NH_3_), hydrogen sulfide (H_2_S), and methane (CH_4_) in the air of the hutch during measurement periods [ppm].

Group		C	D1	D2	D3	SEM	*p*-Value
NH_3_	Measurement period 1	9.36 a	3.18 b	4.30 c	5.55 d	0.086	<0.000
	Measurement period 2	11.89 a	5.74 b	9.31 c	10.07 d	0.080	<0.000
H_2_S	Measurement period 1	ni.	ni.	ni.	ni.	-	-
	Measurement period 2	ni.	ni	ni.	ni.	-	-
CH_4_	Measurement period 1	0.03	0.01	0.01	0.02	0.001	0.270
	Measurement period 2	0.04	0.02	0.03	0.03	0.001	0.180

SEM—standard error of the mean, ni.—not identified, a, b, c, d—for each line values marked with different letters differ statistically significantly at *p* < 0.05.

**Table 2:** Particulate concentrations in the air of the rabbit hutch measurement periods [µg/m^3^].

Group		C	D1	D2	D3	SEM	*p*-Value
PM2.5	Measurement period 1	84.39 a	19.13 b	21.59 b	36.34 c	1.700	<0.000
	Measurement period 2	64.68 a	43.38 b	40.96 c	35.35 d	0.445	<0.000
PM10	Measurement period 1	86.46 a	20.20 b	22.59 b	38.33 c	1.723	<0.000
	Measurement period 2	66.68 a	45.38 b	42.96 c	37.35 d	0.445	<0.000

SEM—standard error of the mean, a, b, c, d—for each line values marked with different letters differ statistically significantly at *p* < 0.05.

**Table 3:** Concentrations of gaseous pollutants in each study period [ppm].

Group	Period	C	D1	D2	D3	SEM	*p*-Value
NH_3_	1	85.36 a	26.66 b	59.09 c	91.47 a	1.05	<0.000
	2	4.11 a	2.98 b	3.82 ac	4.29 acd	0.05	<0.000
	3	2.15 a	1.97 b	2.68 c	2.82 c	0.13	<0.000
	4	2.00 a	1.25 b	2.67 c	2.31 ac	0.074	<0.000
CH_4_	1	4.44 a	3.97 a	3.94 a	3.19 b	0.07	<0.000
	2	3.25 a	2.25 b	2.19 b	2.29 b	0.03	<0.000
	3	2.87 a	2.44 b	2.34 b	2.42 b	0.02	<0.000
	4	2.98 a	2.42 b	2.33 b	2.32 b	0.015	<0.000
H_2_S	1	3.24 a	1.29 b	1.29 b	1.29 b	0.03	<0.000
	2	1.41 a	1.32 b	1.32 b	1.31 b	0.00	<0.000
	3	1.43 a	1.35 b	1.35 b	1.35 b	0.00	<0.000
	4	1.42 a	1.36 b	1.35 b	1.35 b	0.067	<0.000

SEM—standard error of the mean; a, b, c, d—for each line values marked with different letters differ statistically significantly at *p* < 0.05.

## Data Availability

The data presented in this study are available on request from the corresponding author. The data are not publicly available due to the fact that they form part of an ongoing study.
